# Hyperglycemic conditions inhibit C3-mediated immunologic control of ***Staphylococcus aureus***

**DOI:** 10.1186/1479-5876-10-35

**Published:** 2012-03-05

**Authors:** Pamela S Hair, Charlene G Echague, Reuben D Rohn, Neel K Krishna, Julius O Nyalwidhe, Kenji M Cunnion

**Affiliations:** 1 Department of Pediatrics, Eastern Virginia Medical School, 855 West Brambleton Avenue, Norfolk, VA, USA; 2 Children's Specialty Group, 601 Children's Lane, Norfolk, VA, USA; 3 The Children's Hospital of The King's Daughters, 601 Children's Lane, Norfolk, Norfolk, VA 23501-1980, USA; 4 Department of Microbiology and Molecular Cell Biology, Eastern Virginia Medical School, 700 West Olney Road, Norfolk, VA, USA; 5 Leroy T. Canoles Jr. Cancer Research Center, Eastern Virginia Medical School, 700 West Olney Road, Norfolk, Virginia, USA

**Keywords:** Complement C3, *Staphylococcus aureus*, Hyperglycemia, Immune evasion, Polymicrobial infection

## Abstract

**Background:**

Diabetic patients are at increased risk for bacterial infections; these studies provide new insight into the role of the host defense complement system in controlling bacterial pathogens in hyperglycemic environments.

**Methods:**

The interactions of complement C3 with bacteria in elevated glucose were assayed for complement activation to opsonic forms, phagocytosis and bacterial killing. C3 was analyzed in euglycemic and hyperglycemic conditions by mass spectrometry to measure glycation and structural differences.

**Results:**

Elevated glucose inhibited *S. aureus *activation of C3 and deposition of C3b and iC3b on the bacterial surface. *S. aureus*-generated C5a and serum-mediated phagocytosis by neutrophils were both decreased in elevated glucose conditions. Interestingly, elevated glucose increased the binding of unactivated C3 to *S. aureus*, which was reversible on return to normal glucose concentrations. In a model of polymicrobial infection, *S. aureus *in elevated glucose conditions depleted C3 from serum resulting in decreased complement-mediated killing of *E. coli*. To investigate the effect of differing glucose concentration on C3 structure and glycation, purified C3 incubated with varying glucose concentrations was analyzed by mass spectrometry. Glycation was limited to the same three lysine residues in both euglycemic and hyperglycemic conditions over one hour, thus glycation could not account for observed changes between glucose conditions. However, surface labeling of C3 with sulfo-NHS-biotin showed significant changes in the surface availability of seven lysine residues in response to increasing glucose concentrations. These results suggest that the tertiary structure of C3 changes in response to hyperglycemic conditions leading to an altered interaction of C3 with bacterial pathogens.

**Conclusions:**

These results demonstrate that hyperglycemic conditions inhibit C3-mediated complement effectors important in the immunological control of *S. aureus*. Mass spectrometric analysis reveals that the glycation state of C3 is the same regardless of glucose concentration over a one-hour time period. However, in conditions of elevated glucose C3 appears to undergo structural changes.

## Background

It has long been recognized that bacterial infections are more common in diabetic patients, especially *S. aureus *diabetic foot infections [1-3], but also more invasive infections like *S. aureus *endocarditis [4-6]. Limb-threatening infections are frequently polymicrobial with enteric Gram-negative bacteria and *S. aureus *present [7-9]. In 2004, diabetics underwent 71,000 non-traumatic limb amputations (American Diabetes Association). In acute settings, hyperglycemia has been associated with increased risk for bacteremia [10] and increased risk of death from *S. aureus *bacteremia [11]. Understanding how pathogenic bacteria interact with critical host defenses in diabetes is a necessary prerequisite for the development of future prevention and treatment strategies.

The human complement system is a major component of innate immunity and plays a vital role in the control of many bacterial pathogens [12] including *S. aureus *[13-15]. C3 is the central component of the complement system and its activation to C3b is critical for bacterial opsonization and subsequent phagocytosis, generation of the anaphylatoxin C5a [16], and terminal complement cascade activation leading to membrane attack complexes (MAC) that can lyse Gram-negative bacteria [17]. Normal C3 levels in human serum is typically about 1 mg/ml [18].

The impact of hyperglycemia on the complement system remains unclear. Diabetic patients are reported to have elevated levels of circulating C3 and C4, yet decreased ability to fix complement by IgG [19], suggesting impaired classical pathway activation. In an animal model of diabetes using non-obese diabetic (NOD) mice, hyperglycemia resulted in chronic *S. aureus *hindpaw infection and decreased killing of *S. aureus *in a whole-blood assay [20]. However, the role of complement was difficult to assess because the mice were C5-deficient and the whole blood-killing assays used heparin, which inhibits complement activation [21]. C3 has been reported to be slowly susceptible to glycation with only 20% glycation after 48 hours in a hyperglycemic environment [22]. It has also been shown that glucose can bind the biochemically active (thioester) site of C3 resulting in decreased attachment to the surface of microbes [23]. For this to occur however, activation of C3 by the alternative pathway was necessary to expose the reactive thioester [24,25]. It has also been postulated that *S. aureus *may improve the survival and pathogenicity of other organisms in polymicrobial infections in diabetes [26].

To our knowledge, published data do not exist with regards to the concentrations of complement proteins or complement activity in diabetic wounds. Blister fluid generated by burn injury have C3 levels decreased approximately two-thirds compared with serum [27]. Additionally, diabetic microvascular disease diminishes blood flow [28], potentially limiting C3 delivery to diabetic extremities. It is unknown to what extent C3 might be generated locally by macrophages [29] in diabetic wounds. Very limited data suggest that glucose levels in diabetic foot ulcers are decreased 6% compared with arterial levels [30].

The experiments below investigate the functional interaction between complement C3 and pathogenic bacteria in elevated glucose, focusing on *S. aureus*. Additionally, we utilize mass spectrometry to analyze the glycation and tertiary structure of C3 in euglycemic and hyperglycemic environments. In summary, elevated glucose inhibits the normal activation of C3 on the surface of *S. aureus*. Hyperglycemic conditions do not rapidly alter glycation of C3, but does appear to alter the tertiary structure.

## Methods

### Ethics Statement

Human blood was obtained from healthy volunteers for generating serum used as a reagent in these studies. Eastern Virginia Medical School IRB approved this study protocol: 02-06-EX-0216. Written informed consent was provided by study participants.

### Bacteria and growth

*S. aureus *strain Reynolds was grown in 2%NaCl Columbia broth at 37°C to stationary-phase growth [31], unless otherwise noted. A sortase-deficient isogenic mutant [32] was tested against strain Newman. Clinical isolates were obtained as discarded de-identified isolates (EVMS IRB 06-04-WC-0040) - speciation by CLSI criteria [33]. Three isolates were tested for each species: *E. coli*, *E. cloacae*, *K. pneumoniae*, *P. aeruginosa*, and *S. marcescens*. PFGE analysis confirmed non-identical strains.

### Buffers serum and complement components

PBS was supplemented with glucose (D-(+)-Dextrose D9434 Sigma). Glucose concentrations were tested in ranges consistent with euglycemia (3 - 6 mmol/l) [34] and moderate hyperglycemia (10-17 mmol/l) [35]. Complement activation was stopped with EDTA-GVBS^- ^buffer (veronal-buffered saline [VBS] with 0.1% gelatin and 0.01 M EDTA) [31]. Normal human serum (NHS) was prepared from human blood and pooled, as previously described [31]. Purified C3, C3b, and iC3b were purchased (CompTech) and tested for purity and functionality [36].

### Incubation of S. aureus with C3 or serum in glucose

Washed bacteria (1 × 10^9^) were combined with purified C3 (1 μg), C3b, or NHS in 100 μL of PBS/glucose and incubated for 1 hour at 37°C, unless otherwise noted. NHS percentage and glucose concentrations used are shown in each figure. Washed bacteria were stripped of C3fragments using methylamine, as previously described [31]. These amounts of bacteria are consistent with those commonly found in established *S. aureus *infections [37].

### Assays for C3-fragments and C5a

Bound C3 was quantitated by total C3 ELISA. Flat-bottom Immunlon-2 plates were coated with goat anti-human C3 antibody (Complement Technology) at 76 ug/ml in a carbonate buffer overnight at 4°C. Plates were washed three times with PBST (PBS with 0.1% Tween-20) and blocked with 3% BSA/PBST for 2 hours at room temperature. Next, plates were incubated with test samples or pure C3 for use as a standard curve for 1 hour diluted in block buffer. Plates were washed as stated above and incubated with a chicken anti-human C3 antibody at a 1:25000 dilution (Sigma) for 1 hour at room temperature. Finally, plates were washed again and incubated with a goat anti-chicken HRP antibody at 1:1000 for 1 hour at room temperature. Plates were developed with TMB Substrate Solution (Thermo Scientific) and stopped with 2.5 N H2SO4. C3-fragments analyzed by Western blot were probed with antibody that recognizes the peptide chains of C3, C3b and iC3b, as previously described [38]. NHS in PBS/glucose was incubated with 1 × 10^9 ^*S. aureus *for 1 hour at 37°C, sedimented, and measured for C5a using a C5a ELISA kit (R&D Systems). C3a dot-blot quantitation was performed by titrating pure C3a (Complement Technology) and samples onto PVDF. The membrane was blocked with 3% BSA/TBS Tween, probed with rabbit anti-C3a antibody (Complement Technology), washed, incubated with horseradish peroxidase-labeled anti-rabbit antibody (Sigma), washed, and followed by ECL. Optical densitometry measurements of the dots provided grey scale values from which a standard curve was generated and samples were quantitated by linear regression.

### Phagocytosis

Neutrophils were purified from human blood, as previously described [39]. An aliquot of 1 × 10^9 ^*S. aureus*, log-phase, was incubated in 5% NHS in PBS/glucose and incubated for 15 min. at 37°C. An aliquot of bacteria/serum suspension was incubated with neutrophils in HBSS (20:1, bacteria: neutrophil), decreasing the final concentration of glucose to 0.3 mmol/l, and tumbled for 45 min. at 37°C. Samples were fixed by cytospin, stained with acridine orange, quenched with crystal violet, and analyzed as previously described [39].

### Serum complement-mediated killing

2% NHS/PBS supplemented with glucose was incubated with 10^9 ^*S. aureus *for 1 hour at 37°C, then sedimented and the supernatant was sterile filtered. A serum-sensitive *E. coli *isolate was grown to log phase and washed. In a microtiter plate, 100 μl of the *S. aureus*-treated supernatant containing 2% NHS was combined with 10^4 ^*E. coli *and incubated for 3 hours at 37°C. Control samples were processed identically, but without serum. Bacteria were quantitated by colony counting.

### Mass spectrometry analysis of C3

Purified C3 (0.4 mg/ml) was incubated in PBS with concentrations of glucose (0, 3 mM, 6 mM, 10 mM, or 17 mM) for 1 hour at 37°C. The samples were then biotinylated using the EZ-Link Sulfo-NHS-Biotin kit (Pierce, Rockford, IL) at a molar ratio of 10 biotins per lysine residue of C3. Excess biotin was removed by spin column and samples were separated by SDS-PAGE. C3 gel bands were excised and digested with trypsin and the extracted peptides were analyzed by ESI-LC MS/MS in a LTQ linear ion trap (Thermo Fisher) mass spectrometer using data dependent acquisition and dynamic exclusion, similar as described previously [40]. Normalized peptide extracts were automatically loaded on a CapTrap column (TR1/25109/32 C18; Michrom Bioresources Inc) via an autosampler, followed by chromatographic separation under the following conditions: Solvent A (0.1% formic acid, 0.005% HFBA) and Solvent B (95% acetonitrile in 0.1% formic acid, 0.005% HFBA). The tryptic digests were eluted at 500 nl/min with PicoFrit columns (75 μm inner diameter, 2 μM tip opening, New Objective, Woburn, MA) slurry-packed in house with 10 cm of reverse phase 5 μm 100 Angstrom Magic C18 resin (Michrom Bioresources, Auburn, CA). The acquisition cycle consisted of a survey MS scan with a set mass range from 350 m/z to 1800 m/z at the highest resolving power, followed by 5 data-dependent MS/MS scans using collision dissociation fragmentation (CID) method assisted with helium gas. Dynamic exclusion was used with the following parameters: exclusion size 50, repeat count 3, repeat duration 120 s, exclusion time 180 s, exclusion window ± 0.8 Da. Target values were set at 5 × 10^5 ^and 10^4 ^for the survey and Tandem MS scans, respectively, and the maximum ion accumulation times were set at 200 ms in both cases. Regular scans were used both for the precursor and tandem MS with no averaging. In total, 15 MS runs were performed with extensive blanks between each sample to avoid carry-over of peptides that could bias quantification. Peak lists were generated using XCalibur (version 2.1). Sequence analysis was performed with MASCOT (version 2.2.03) using SwissProt 2010 × (SwissProt 57.1) database with a human taxonomy filter enabled that contained 516, 603 sequences entries. The database searches were performed with fixed modification as carbamidomethyl (C) and variable modifications as oxidation (M), deamidation (N, D) phosphorylation (STY) and biotin labeled (K). Enzyme specificity was selected to trypsin with 2 missed cleavage sites. The mass tolerance was set at 0.8 Da for both precursor ions and fragment ions. Threshold score for acceptance of individual spectra was set at 0.05. All the MS/MS spectra were manually inspected to verify the validity of the database search results. False discovery rates were estimated to be 0.25% on the protein level by searching a decoy version of the SwissProt protein database.

Relative protein quantitation was achieved by comparing the number of MS/MS spectra for the same protein between the three MS/MS analyses for each sample. An increase in protein abundance is directly related to the number of proteolytic peptides. An increase in the number (or abundance) of peptides increases the number of MS/MS generated. There is a linear correlation between spectral counts and relative protein abundance (R^2 ^= 0.9997) over 2 orders of magnitude [41]. Quantitation via spectral counting shows strong correlation with isotopic label based approaches such as ^14^N/^15^N [42] and precursor peak area intensity measurements [43]. Spectral count based quantitation has been widely applied across a diverse set of media [44]. In the current analyses, biotinylation of lysine residues in C3 after exposure to increasing concentrations of glucose was determined by spectral counting as follows. The sulfo-NHS-biotin derivative reacts with the epsilon amino group of lysine residues thereby increasing the mass of the modified lysine residues by 226 Da. Glycated lysines were identified by the increase in mass of 162 Da due to the addition of a hexose molecule.

Scaffold (Proteome Software, Portland, Oregon, USA) was used to validate protein identifications derived from MS/MS sequencing results. Scaffold verifies peptide identifications assigned by Mascot using the X! Tandem database search program. Scaffold then probabilistically validates these peptide identifications using PeptideProphet and derives corresponding protein probabilities using ProteinProphet. The relative abundance of biotin modified lysine residues/peptides was obtained by spectral counting using Scaffold Q software. Scaffold normalizes these data by averaging the spectrum counts for all of the bio-samples and then multiplying the spectrum counts in each sample by the average divided by the individual sample's sum [45].

### Statistical analysis

Means and standard errors were calculated from independent experiments (Microsoft Excel XP). Statistical comparisons were made using Student *t *test (Instat GraphPad). Linear regression analyses (InStat GraphPad) were performed for spectral counts relative to glucose concentration.

### C3 structural models

Three-dimensional drawings of C3 was generated using PyMOL software http://www.pymol.org based on the reported crystallographic structure of C3 [46].

## Results

### Glucose inhibition of C3-mediated opsonophagocytosis of S. aureus

**Figure 1:**
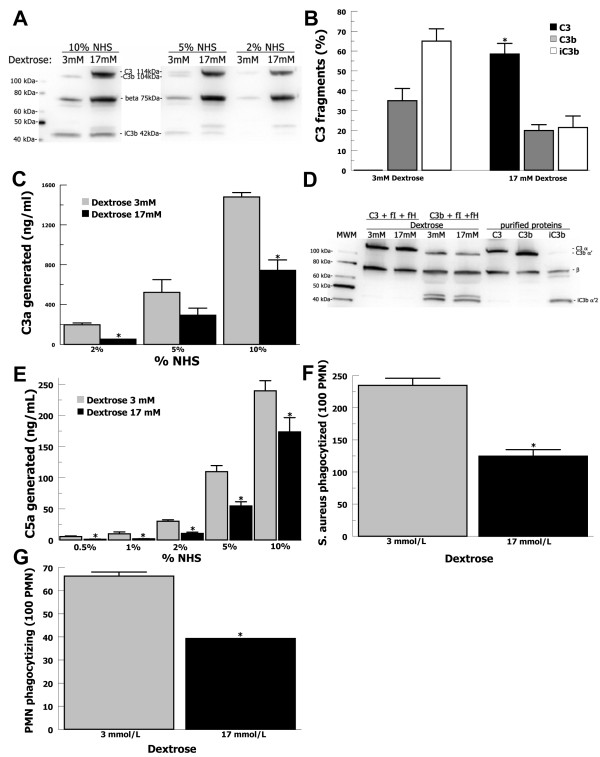
**C3-mediated opsonophagocytosis of *S. aureus *in elevated glucose**. (**A**) C3 binding to *S. aureus *in 2%, 5%, or 10% NHS in PBS with 3 mmol/l glucose or 17 mmol/l glucose assayed by Western blot. (**B**) Optical densitometry measurements of the forms of C3 (C3, black bars; C3b, grey bars; iC3b, white bars) present on the *S. aureus *surface in 10% NHS for 3 mM or 17 mM glucose. Alpha-chain densitometry was normalized to the beta-chain and shown as a total percent of C3 forms. Data are mean ± SE for 4 independent experiments. (**C**) C3a generation by *S*. aureus in varying serum and glucose concentrations (3 mmol/l, grey bars and 17 mmol/l, black bars) was assayed by dot blot. Data are mean ± SE for 3 independent experiments. (**D**) Purified C3 incubated with purified factor H (fH) and factor I (fI) shows no significant cleavage in 3 or 17 mmol/l glucose, whereas expected cleavage occurs for purified C3b in these conditions. (**E**) For *S. aureus *incubated in various concentration of NHS, C5a generation was increased in 3 mmol/l glucose (grey bars) compared with 17 mmol/l glucose (black bars). Data are mean ± SE for 4 independent experiments. (**F**, **G**) *S. aureus *opsonized in 5% NHS in 3 mmol/l glucose (grey bars) showed increased phagocytosis by human neutrophils compared with bacteria opsonized in 17 mmol/l glucose (black bars). The number of bacteria phagocytized by 100 PMN and the number of neutrophils phagocytizing bacteria were counted by fluorescence microscopy. Data are mean ± SE for 3 independent experiments.

In order to test whether hyperglycemic conditions altered C3 activation and binding of activated C3-fragments (C3b and iC3b) on the *S. aureus *surface, we performed Western blot analysis of C3-fragments stripped from *S. aureus *incubated in serum in euglycemic, 3 mmol/l dextrose, and hyperglycemic, 17 mmol/l dextrose, conditions (Figure 1A). In each serum concentration tested, more C3 beta-chain was present for the 17 mmol/l glucose condition compared with the 3 mmol/l glucose condition, suggesting that more C3 was binding to the *S. aureus *surface in elevated glucose over 1 hour. However, for the 17 mmol/l condition most of the C3 alpha-chain appears to be intact (114 kDa) [29] and uncleaved suggesting that most of the C3 binding *S. aureus *in elevated glucose is not being activated to C3b. As expected, in normal glucose conditions (3 mmol/l glucose), C3 is activated efficiently with minimal or no intact C3 alpha-chain present, resulting in the 104 kDa α' fragment (C3b) and the 42 kDa α'2 (iC3b) bound to the *S. aureus *surface. We then performed optical densitometry on Western blots from multiple independent experiments in 10% NHS in both glucose conditions (Figure 1B). C3 alpha-chain densitometry was normalized to the invariant beta-chain, as previously described [47]. In 3 mmol/l glucose only C3b (35%) and iC3b (65%) were bound to the *S. aureus *surface with no intact C3 identifiable. In 17 mmol/l intact C3 was most prevalent (59%) compared with C3b (20%) and iC3b (21%) (P = 0.002). In order to further test whether C3 activation by *S. aureus *was altered in elevated glucose we measured C3a generation by dot-blot analysis for varying concentrations of serum (Figure 1C). C3a generation was increased in 3 mmol/l glucose compared with 17 mM glucose by 2-fold (P = 0.01) in 10% NHS and 3-fold (P = 0.01) in 2% NHS. In order to evaluate for the possibility of C3(H2O) being generated in hyperglycemic conditions, we tested whether C3 in 3 or 17 mmol/L glucose would be cleaved by the presence of purified factors H and I. C3 remained intact in conditions that efficiently cleaved C3b to iC3b suggesting that hyperglycemia was not generating C3(H2O) (Figure 1D). These findings suggested that C3 activation on the *S. aureus *surface was inhibited in elevated glucose thereby limiting deposition of C3b/iC3b opsonins.

Limited generation of C3b on the *S. aureus *surface would be expected to decrease the formation of C5-convertases and generation of the potent anaphylatoxin C5a [29]. *S. aureus *was incubated in serum in PBS/glucose and C5a generation was measured by ELISA (Figure 1E). In 17 mmol/l glucose, C5a generation was decreased 5-fold (P = 0.04) in 1% NHS and decreased 2-fold (P < 0.01) in 5% NHS. This suggested that elevated glucose inhibited serum complement activation by *S. aureus *which decreased anaphylatoxin generation, likely resulting from decreased activation of C3 on the staphylococcal surface.

Decreased deposition of C3b/iC3b on *S. aureus *and decreased generation of C5a in the presence of elevated glucose suggested that phagocytosis would also be inhibited. Thus, *S. aureus *were incubated in 5% NHS for 15 minutes in PBS/glucose. A sample of this bacteria/serum suspension was added to a suspension of purified neutrophils, diluting the concentration of glucose to 0.3 mmol/l, and incubated for 45 minutes. The number of *S. aureus *phagocytized after opsonization in 17 mmol/l glucose was decreased by 2-fold (P < 0.01) compared with 3 mmol/l glucose (Figure 1F) and the number of neutrophils phagocytizing bacteria was likewise decreased (P < 0.01) in 17 mmol/l glucose (Figure 1G). The concentration of glucose was diluted upon addition of bacteria to neutrophils to minimize glucose effects on neutrophil function. It is possible, however, that decreasing the glucose concentration could have increased complement activation during incubation with neutrophils resulting in diminished differences in phagocytosis efficiency. Nonetheless, these results suggest that elevated glucose inhibited complement-mediated opsonophagocytosis of *S. aureus*.

### Glucose concentration affects C3 binding to S. aureus without C3-convertase formation

**Figure 2:**
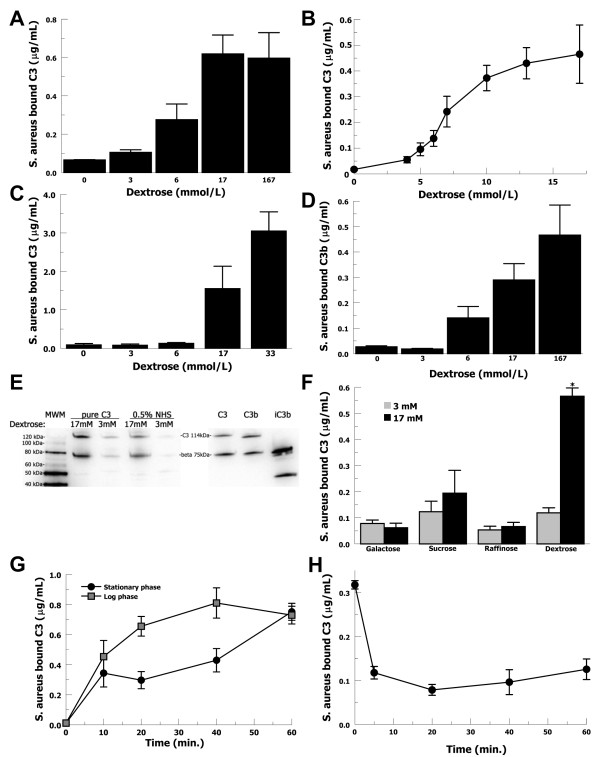
**Purified and serum C3 binding to *S. aureus *in varying glucose concentrations**. (**A**, **B**) Purified C3 (10 μg/ml) binding to *S. aureus *in PBS increases at elevated levels of glucose. Data are mean ± SE for 4 independent experiments. (**C**) C3 binding to *S. aureus *incubated in 0.5% NHS in EDTA-GVBS^- - ^buffer, to prevent complement activation, increases at elevated glucose concentrations. Data are mean ± SE for 4 independent experiments. (**D**) Purified C3b binding to *S. aureus *in PBS increased at elevated levels of glucose. Purified C3b is incapable of activation. Data are mean ± SE for 4 independent experiments. (**E**) C3 bound to *S. aureus *incubated with purified C3 or 0.5% NHS in PBS was increased for 17 mmol/l glucose compared with 3 mmol/l. Purified standards for C3 [alpha (114 kDa) and beta (75 kDa) chain and iC3b products α2 ^' ^(42 kDa)] were included on the gel. (**F**) Purified C3 binding to *S. aureus *in PBS did not increase in 17 mmol/l (black bars) compared to 3 mmol/l (gray bars) for galactose, sucrose, or raffinose. Data are mean ± SE for 4 independent experiments. (**G**) Purified C3 binding to stationary phase (black circles) or log phase (grey squares) *S. aureus *in elevated glucose (17 mmol/l) increased over time. Data are mean ± SE for 3 independent experiments. (H) *S. aureus *bound by C3 in elevated glucose (17 mmol/l) and then incubated in 3 mmol/l glucose shows a rapid decrease for residual C3 bound to the bacteria. Data are mean ± SE for 3 independent experiments.

Initial Western blot analysis suggested that intact C3, without activation to C3b, was binding to *S. aureus *in elevated glucose conditions. In order to evaluate whether C3 binding could be replicated in the absence of other complement components, thus preventing convertase formation, we measured purified C3 binding to *S. aureus*. Bacteria were incubated for 1 hour in PBS/glucose with purified C3 (10 μg/ml). C3 was stripped from washed bacteria and measured by ELISA (Figure 2A). In elevated glucose (17 mmol/l), C3 binding to *S. aureus *increased 6fold (P < 0.01) compared with 3 mmol/l glucose. C3 binding rapidly increased above 5 mmol/l and reached saturation at 13 - 17 mmol/l (Figure 2B). Similar binding studies were performed with purified C3 and *S. aureus *in GVBS^++ ^and VBS buffers with similar results (data not shown). In order to evaluate serum C3 binding, but still prevent complement activation, bacteria were incubated with 0.5% NHS in EDTA-GVBS^- - ^buffer supplemented with glucose (Figure 2C). In the presence of elevated glucose (17 mmol/l), serum C3 binding increased 12-fold (P = 0.049) compared with 6 mmol/l glucose. In order to test the contribution of anti-staphylococcal antibodies, we depleted these antibodies from serum under complement preserving conditions, as previously described [38]. The binding of intact C3 to *S. aureus *in hyperglycemic conditions was the same for specific antibody-depleted serum compared with NHS (data not shown). Overall, greatly increased C3 binding to *S. aureus *is found in elevated glucose concentrations similar to what would be found in moderate hyperglycemia (10-17 mmol/l) [35] as compared to euglycemia (3 - 6 mmol/l).

In order to exclude the possibility of spontaneous non-convertase-dependent C3 activation, we tested the binding of purified C3b to *S. aureus *(Figure 2D). Purified C3b is no longer capable of activation and cannot form covalent bonds. Bound C3b increased 14-fold for 17 mmol/l glucose compared with 3 mmol/l glucose (P = 0.025). Western blot analysis confirmed minimal C3 binding in 3 mmol/l glucose and that intact C3 bound in 17 mmol/l glucose (Figure 2E).

In order to evaluate whether glucose-mediated C3 binding to *S. aureus *is caused by the high osmolarity environment, we tested multiple sugars at the same osmolarities. *S. aureus *was incubated with purified C3 and galactose, sucrose, and raffinose at 3 mmol/l and 17 mmol/l, but C3 binding remained unchanged (Figure 2F), suggesting a mechanism other than osmolarity.

In order to evaluate the rapidity of glucose-mediated C3 binding to *S. aureus*, we performed time-course experiments. We also evaluated different phases of growth as this affects the surface expression for some *S. aureus *proteins [48]. *S. aureus *in stationary or mid-logarithmic phase was incubated with purified C3 in PBS at elevated glucose levels (17 mmol/l) for increasing lengths of time (Figure 2G) and measured for C3 binding. C3 initially bound more slowly to stationary phase *S. aureus *compared with mid-logarithmic phase bacteria, but by 60 minutes the same amount of C3 was bound.

Because C3 appears to be binding *S. aureus *in elevated glucose without activation and formation of covalent bonds, we wanted to assess whether glucose-mediated C3 binding to *S. aureus *would be reversed in a euglycemic environment. Purified C3 was allowed to bind to *S. aureus *in 17 mmol/l glucose, washed in PBS/glucose (3 mmol/l), and then incubated in PBS/glucose (3 mmol/l) (Figure 2H). C3 bound to *S. aureus *decreased during washing in 3 mmol/l glucose and after 5 minutes the amount of bound C3 decreased a further 3-fold (P = 0.005). This suggested that glucose-mediated C3 binding to *S. aureus *is readily reversible upon return to euglycemic conditions.

### C3 binding to clinical S. aureus and Gram-negative isolates

**Figure 3:**
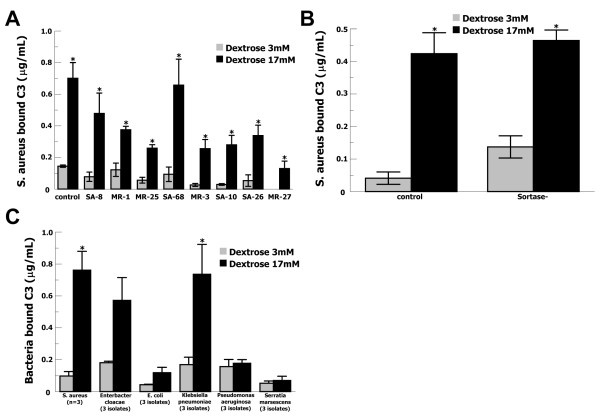
**C3 binding to clinical isolates of pathogenic species and mutant *S. aureus *in elevated glucose**. (**A**) Purified C3 binding to 8 clinical *S. aureus *isolates was increased in 17 mmol/l glucose (black bars) compared with 3 mmol/l glucose (grey bars). Four strains are MRSA (MR) and four strains are methicillin-susceptible (SA). Data are mean ± SE for 3 independent experiments for each isolate. Strain Reynolds was used as a control. (**B**) Purified C3 binding to a sortase-deficient *S. aureus *strain (Sortase-) and an isogenic control was similarly increased in 17 mmol/l glucose (black bars) compared with 3 mmol/l glucose (grey bars). Data are mean ± SE for 3 independent experiments. (**C**) Purified C3 binding to 15 clinical Gram-negative isolates in PBS with 3 mmol/l glucose (grey bars) or 17 mmol/l glucose (black bars). Three isolates were tested for each species; data are mean ± SE.

In order to evaluate whether glucose-meditated C3 binding to *S. aureus *is a general property of clinical *S. aureus *strains, we tested 8 clinical isolates (Figure 3A). All isolates were recovered from patients with invasive *S. aureus *disease; four isolates were methicillin-resistant (MRSA). Isolates were non-identical by pulse-field gel electrophoresis. For each isolate, elevated glucose concentration increased C3 binding significantly (P ≤ 0.05). On average, 17 mmol/l glucose increased C3 binding 6-fold (P = 0.001), suggesting that glucose-mediated C3 binding to *S. aureus *is a general property of clinical isolates.

We speculated whether glucose-mediated C3 binding to *S. aureus *could be mediated by LPXTG-motif anchored cell-wall proteins. Therefore, we tested a sortase-deficient *S. aureus *strain [32] and its isogenic parent strain, Newman (Figure 3B). C3 binding in 17 mmol/l glucose was the same for both the parental strain and the sortase-deficient strain, suggesting that LPXTG-motif cell-wall proteins do not mediate this phenomenon.

In order to evaluate whether glucose-mediated C3 binding occurred for other bacterial species, we tested 15 Gram-negative clinical isolates (Figure 3C). Three isolates were tested for each of five species. Pulse-field gel electrophoresis testing demonstrated that strains were non-identical. Significantly increased C3 binding in 17 mmol/l glucose was found for *K. pneumoniae *(P = 0.04), but not *P. aeruginosa*, or *S. marcescens*. *E. cloacae *(P = 0.07) and *E. coli *(P = 0.07) strains suggested a trend towards significance. These results show that glucose-mediated C3 binding to bacteria also occurs for at least one Gram-negative species, but is not a generalized property for all bacterial species.

### C3 depletion from the local environment and complement killing of E. coli

**Figure 4:**
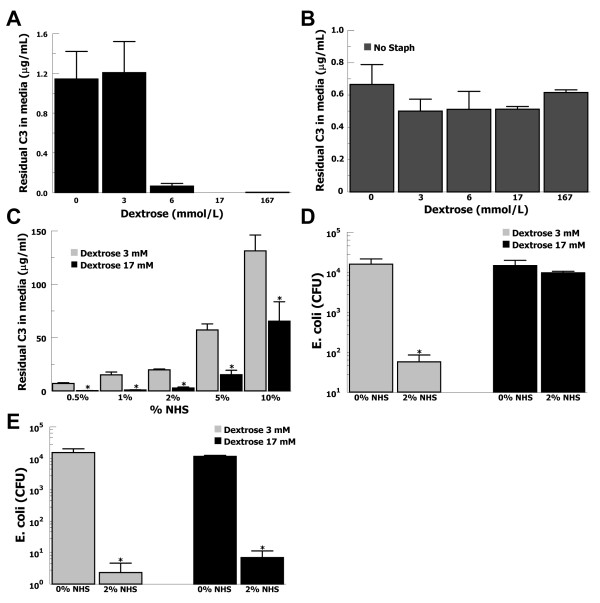
**C3 depletion by *S. aureus *in elevated glucose is associated with decreased complement-mediated killing of *E. coli***. (**A**) At elevated glucose levels, purified C3 is depleted from buffer in the presence *S. aureus*. Data are mean ± SE for 4 independent experiments. (**B**) In the absence of *S. aureus*, purified C3 is not depleted from buffer. Data are mean ± SE for 4 independent experiments. (**C**) *S. aureus *incubated with varying concentrations of NHS shows decreased residual C3 in the supernatant for 17 mmol/l glucose (black bars) compared with 3 mmol/l glucose (grey bars). Data are mean ± SE for 4 independent experiments. (**D**) For 2% NHS pre-incubated with *S. aureus *at 3 mmol/l glucose (grey bars) surviving *E. coli *decreased > 2 logs compared with 0% NHS. For 2% NHS pre-incubated with *S. aureus *at 17 mmol/l glucose (black bars) there was no difference in *E. coli *survival compared with 0% NHS. Data are mean ± SE for 4 independent experiments. (**E**) Control experiments performed without *S. aureus *in the preincubation step showed similar killing of *E. coli *at 3 mmol/l glucose (grey bars) or 17 mmol/l glucose (black bars). Data are mean ± SE for 3 independent experiments.

In light of the dramatic increases in C3 binding to *S. aureus*, we speculated that *S. aureus *in high glucose concentrations might deplete C3 from the local environment. C3-depletion could potentially play an important role in diabetic wound infections where C3 would likely be present in limited quantities. *S. aureus *was incubated with purified C3 (10 μg/mL) as above, sedimented, and supernatants measured for residual C3 by ELISA (Figure 4A). In conditions of elevated glucose (17 mmol/l), residual C3 concentrations decreased 10-fold (P < 0.01) compared with 3 mmol/l glucose. Using identical conditions, but without *S. aureus*, no differences in residual C3 concentration were found between any glucose concentrations (Figure 4B), showing that the C3 was not being lost by precipitation or binding to the wall of the reaction tube.

We then assayed whether C3 would be depleted from serum incubated with *S. aureus *in elevated glucose (Figure 4C). In 10% serum, residual C3 decreased 2-fold (P = 0.05) for 17 mmol/l glucose compared with 3 mmol/l glucose. These results suggest that *S. aureus *may deplete C3 in local environments where C3 is present in limiting concentrations and glucose concentration is elevated, such as in diabetic wounds.

Given that C3 can be depleted from the local environment by *S. aureus *in elevated glucose, we speculated that Gram-negative bacteria susceptible to complement-mediated lysis could incur a survival advantage in polymicrobial infections. Such an effect could greatly worsen the disease process in limb-threatening diabetic wound infections in which *S. aureus *and Gram-negative bacteria are commonly present [8]. *S. aureus *was incubated in PBS/glucose ± 2% serum for 1 hour and then removed from the solution. The serum solutions were then incubated with a serum-sensitive strain of *E. coli *for three hours and plated for colony counting (Figure 4D). In 3 mmol/l glucose, the number of surviving *E. coli *in 2% NHS was decreased > 200-fold (P = 0.03) compared with no serum, typical of complement-mediated lysis. However, in 17 mmol/l glucose, there was no difference in the numbers of surviving *E. coli *between 2% NHS and the no serum control. These results suggest that glucose-mediated depletion of C3 by *S. aureus *can inhibit complement-mediated lysis of Gram-negative bacteria improving their survival.

In order to test whether elevated glucose might prevent complement-mediated lysis of the *E. coli*, we repeated the previous experiments excluding *S. aureus *from the pre-incubation step (Figure 4E). This resulted in similar levels of complement-mediated killing of *E. coli *in 17 or 3 mmol/l glucose, suggesting that elevated glucose did not inhibit complement-mediated killing of *E. coli*.

### Mass spectrometry analysis of C3 glycation and structure in elevated glucose

**Figure 5:**
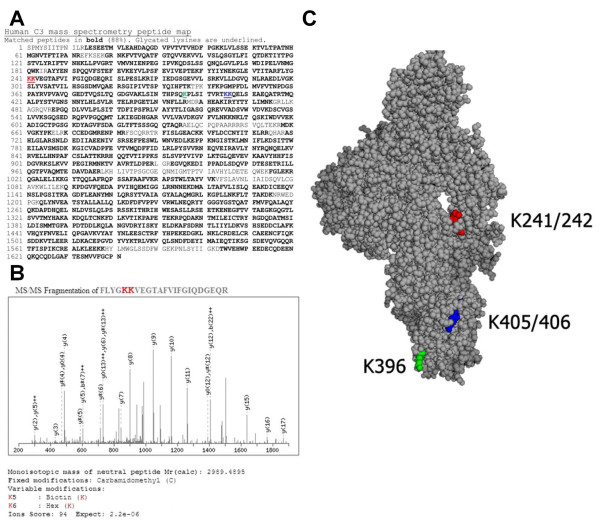
**Mass spectrometry analysis of C3 glycation in changing glucose**. (**A**) A cumulative peptide map of the fifteen C3 samples analyzed by ESI-LC-MS/MS showing 88% coverage. Matched peptides are in bold and glycated residues are underlined. (**B**) A representative mass spectra of the C3 peptide containing K241/K242 showing glycation and biotinylation. (C) A space filling model of C3 showing the positions of glycated lysine residues in color.

In order to evaluate whether the observed changes in C3 interaction with bacteria could potentially result from elevated glucose altering the C3 molecule, we used mass spectrometry-based techniques to evaluate the glycation and tertiary structure of C3. Purified C3 was incubated in either 0, 3, 6, 10, or 17 mmol/l glucose for 1 hour in PBS buffer and then modified by surface biotinylation. Each of these conditions was repeated in three independent experiments. After removal of excess biotin, the C3 molecules were digested to peptides and analyzed by ESI-LC-MS/MS. Typical peptide map coverage for each experiment was excellent at ≥ 61% yielding a cumulative peptide coverage of 88% (Figure 5A). Glycation analysis was performed for C3 for each glucose concentration, yet regardless of the glucose concentration tested (3 - 17 mmol/l) lysines K241/K242, K396, and K405/K406 were consistently glycated. Resolution was not sufficient to differentiate between glycation of K241 and K242 or K405 and K406. Glycation of a lysine prevents subsequent biotinylation. A representative spectrum for the peptide sequence FLYGKKVEGTAFVIFGIQDGEQR containing K241/K242 is shown in Figure 5B. The glycated lysines all reside in the C3 beta chain and are shown superimposed on a structural model of C3 [46] (Figure 5C). These assays show that C3 is readily glycated at these lysine positions, even in normal glucose concentrations, and that over this time frame increasing concentrations of glucose do not alter the glycation pattern. This suggests that glycation is not responsible for changes in C3 interactions in hyperglycemia.

**Figure 6:**
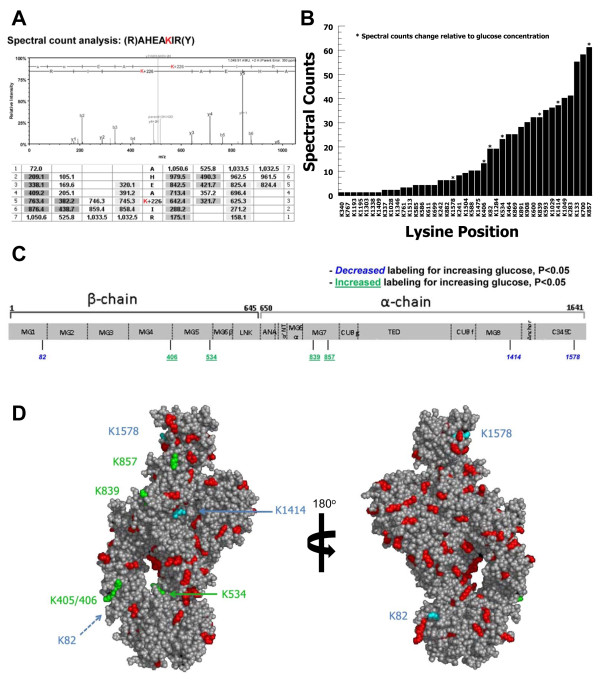
**Mass spectrometry analysis of C3 structure in changing glucose**. (**A**) A representative spectral count analysis of the biotinylation of the C3 peptide AHEAKIR containing K464. (**B**) Cumulative spectral counts for biotin surface modification for each of 41 lysine positions. Lysine residues that showed significant change (P < 0.05) in biotinylation correlating with changing glucose are marked with an asterisk. (**C**) A linear peptide map showing the domains of C3 and the relative positions of the lysines that demonstrated changing biotinylation. Lysines that showed decreased labeling as glucose increased are in italics and lysines that showed increased labeling as glucose increased are underlined. (**D**) Model of C3 showing the positions of lysine residues that were increasingly modified as glucose increased (green) and lysine residues that were decreasingly modified as glucose increased (blue). Other lysine residues are shown in red. 180 degree rotation of model is illustrated to show all lysines.

In order to evaluate whether changes in glucose concentration could alter the tertiary structure of C3, we performed surface modification using sulfo-NHS-biotin. Lysines on the surface of the molecule are more easily modified with biotin, whereas internal lysines are less available for modification. Thus, any alterations in the tertiary structure of the molecule would result in changing the availability of specific lysine residues for biotinylation. For the 15 samples tested in this analysis, 41 lysines were identifiable in every sample. Biotinylation increases the peptide mass by 226 Da which is detected by MS and the specific modified residue is identified by MS/MS. A representative spectrum for the fragmentation and annotation for the C3 peptide sequence AHEAKIR is shown in Figure 6A. The total sequence coverage achieved in the MS analysis to identify the peptides and modified lysine residues was 88%. Most of the peptides that were not identified in the analyses have m/z that fall out of the range of the MS instrument used. Spectral count analysis was performed to quantitate the frequency of biotinylation for each of the 41 lysine residues (Figure 6B). Spectral count analysis allows for the flexibility of molecules in physiological conditions in contrast to crystallized molecules which are static. Sixteen lysines were infrequently labeled with ≤ 5 spectral counts total over 15 samples. These lysine residues were highly resistant to biotinylation for all conditions suggesting that they are not available for surface modification. Interestingly, seven lysine residues showed a statistically significant change in biotinylation relative to changing glucose concentration, as shown in Table 1. Increasing glucose concentration correlated with increasing biotinylation for K405/406, K534, K839, and K857, but decreasing biotinylation for K82, K1414, and K1578. The differentially labeled lysines in the domains of C3 [46] are shown in Figure 6C. The positions of the changing lysines are shown in three-dimensional models in Figure 6D. These results strongly suggest that the tertiary structure of C3 changes as glucose concentration changes and may contribute to altering the interaction of C3 with bacteria in hyperglycemic conditions.

**Table 1:** Lysine residue spectral counts by dextrose concentration

			Dextrose				
**Lysine**	**0 mM**	**3 mM**	**6 mM**	**10 mM**	**17 mM**	**Total**	**P**

K82	6	5	4	2	2	19	0.0264

K405/406	2	2	2	3	4	13	0.0164

K534	2	1	5	6	9	23	0.0242

K839	5	6	6	7	8	32	0.0011

K857	9	12	12	13	15	61	0.0110

K1414	9	8	8	6	6	37	0.0286

K1578	2	2	1	1	0	6	0.0163

In order to more closely examine potential changes in the structure of C3 near the activating cleavage site Arg 726 - Ser 727, we analyzed the mass spectrometry data using threshold criteria to determine whether biotinylation of each lysine between regions MG6β and MG6α was detected for each sample. This approach was used due to the limited spectral count data available for the lysines in this region. Biotinylation was detected for seven lysines in this region, three of which suggested changes in labeling between euglycemic and hyperglycemic conditions (Table 2 suggesting structural changes are likely occurring near the activating cleavage site, which may contribute to decreased C3 activation by pathogenic bacteria in hyperglycemic conditions.,). Two of these lysines (K585/586 and K611) were in MG6β and one (K757) was in MG6α

**Table 2:** Number of experiments for which lysine labeling was detected between C3 regions MG6β and MG6α

		Euglycemia	Hyperglycemia
**Lysine**	**C3 region**	**3 mM and 6 mM glucose**	**10 mM and 17 mM glucose**

K585/586	MG6β	3	0

K588	MG6β	4	5

K593	MG6β	5	6

K600	MG6β	6	5

K611	MG6β	0	2

K700	ANA	6	5

K757	MG6α	0	3

We had circular dichroism performed by Alliance Protein Laboratories (Camarillo, CA). Only very slight differences were noted in the CD patterns for the two conditions (data not shown). This is likely due to the relative insensitivity of CD to detect subtle changes in the tertiary structure of large multi-domain molecules. Two prior publications show that harsh denaturing conditions are required to induce conformational changes in C3 that are detectable by CD [49,50]. Another publication suggests circular dichroism is relatively insensitive in detecting structural changes in large multi-domain molecules [51]. C3 is both large and complex with 1,641 residues and 13 domains [46].

## Discussion

Decreased C3 activation by *S. aureus*, as measured by C3a generation, and decreased deposition of C3b and iC3b on the bacterial surface in elevated glucose strongly suggest that, at least in complement limited environments, hyperglycemic environments inhibit efficient C3-mediated opsonization of *S. aureus*. As expected, diminished deposition of C3b/iC3b resulted in decreased phagocytosis by neutrophils and decreased C3b resulted in decreased C5a generation. Since the normal functioning of neutrophils is already impaired by elevated glucose [52,53], it is likely that inefficient opsonization further retards phagocytosis. Thus, hyperglycemic conditions inhibit efficient complement-mediated opsonophagocytosis and generation of anaphylatoxin, likely contributing to diabetic immunocompromise manifested by frequent infections that are difficult to control.

It is interesting to note that use of continuous insulin infusion in the intensive care setting in order to rapidly correct hyperglycemia has been associated with improved survival [54], which may be due in part to the elevated risk of bacteremia in hyperglycemia [55]. We show that the binding of unactivated C3 is rapidly reversed upon return to euglycemic conditions suggesting that the abnormal interaction of C3 and bacteria can be reversed upon correction of hyperglycemia. We speculate that normalization of the interaction of C3 with bacteria by rapidly reversing hyperglycemia in critical care settings, where risks of invasive infection are elevated, may contribute to the improved survival.

Another important infectious complication for diabetics are polymicrobial limb-threatening infections that commonly involve *S. aureus *and Gram-negative bacteria [7]. Gram-negative bacteria, unlike Gram-positive bacteria, are often susceptible to complement-mediated killing via membrane attack complex formation and lysis. Given the limited levels of complement proteins likely to be present in diabetic wounds, we speculated that *S. aureus *might be able to further deplete C3 in such an environment and confer a survival advantage to Gram-negative organisms. These studies showed that *E. coli *killing by complement-mediated lysis was eliminated by the pre-incubation of serum with *S. aureus *in 17 mmol/l glucose. This suggests that glucose-mediated C3 adsorption to *S. aureus *and depletion from the local environment can dramatically improve survival of Gram-negative bacteria from complement-mediated lysis and may contribute to the pathogenesis of limb-threatening polymicrobial infections.

Prior studies using biochemical methods have suggested that glycation of C3 in high concentrations of glucose is a very slow process [22], which is inconsistent with the rapid functional effects found in these studies. These new mass spectrometry data show that three lysine sites are glycated by 1 hour, but that the glycation results were the same regardless of euglycemic or hyperglycemic conditions. This is novel data delineating the sites where C3 is rapidly glycated, K241/K242, K396, and K405/K406. Since hyperglycemic conditions do not change the glycation compared with euglycemic conditions over short time frames, glycation is unlikely to account for the rapid (< 1 hour) changes in interaction with bacteria in elevated glucose. Prior studies have also suggested that upon activation by the alternative pathway, glucose can bind the critical thioester site of C3 [24]; however, our studies suggest that very limited amounts of C3 is activated on the *S. aureus *surface in the presence of elevated glucose. None of the lysines glycated in our assays reside within the thioester-containing domain (residues 963 - 1268) [46], suggesting that this was not an important mechanism over short time intervals.

We speculated that hyperglycemic conditions must either alter the C3 molecule or a component or components of *S. aureus*. Since glycation of C3 did not change in elevated glucose compared with euglycemic conditions, we tested whether increasing glucose concentration could change the conformation of C3. We assayed for this possibility by using mass spectrometry-based surface modification analysis of lysine biotinylation. Employing spectral count analysis provided quantitative measurement about how frequently a given lysine site was biotinylated. Of 41 lysines analyzed, over one-third were highly resistant to surface modification for all glucose conditions, suggesting that they occupy internalized or protected positions within the C3 molecule. Seven lysines were shown to undergo significant changes in surface modification relative to increasing glucose concentration, suggesting that these regions of the C3 molecule are becoming more or less surface exposed as glucose changes. The changing availability for surface modification suggests that the tertiary structure of C3 is changing in response to changing glucose concentration. The positions of the lysines with changing modification are distributed over the molecule suggesting that multiple areas of the molecule are undergoing structural alteration. These structural changes likely contribute to the altered interaction of C3 with bacteria in hyperglycemic environments. A recent study showed that high glucose inhibited the lectin pathway by inhibiting oligosaccharide recognition, but classical and alternative complement pathway activation were not inhibited in Weislab ELISA-style complement activation assays [56]. We confirmed in CH50 and AP50 assays that serum complement-mediated hemolysis was not altered by elevated glucose (data not shown). This suggests that changes in the structure of C3 likely contribute to the dramatically altered interaction with pathogenic bacteria, but are not so great as to globally cripple the ability of the molecule to function. Together with the rapid reversibility of functional effects upon return to euglycemic conditions suggests that the structural alterations are likely to result from non-covalent changes. It also remains undetermined whether hyperglycemic environments may alter the surface of pathogenic bacteria, as has been shown for yeast [57], contributing to the altered interaction with C3.

## Conclusions

The principal finding of our study is that in conditions of elevated glucose, activation of complement C3 to functionally active forms (C3b/iC3b) on the *S. aureus *surface was inhibited. Curiously, elevated glucose caused the reversible binding of unactivated C3 to *S. aureus*. The limited activation of C3 on the *S. aureus *surface in elevated glucose was associated with decreased C5a generation and decreased phagocytosis. Mass spectrometric analysis demonstrated that the glycation of C3 was the same for euglycemic and hyperglycemic conditions over brief time periods and thus, could not account for the rapid changes in function. Mass spectrometry-based surface labeling experiments suggested that the tertiary structure of C3 was altered in the presence of increasing glucose. Thus, hyperglycemic conditions dramatically alter the interaction of C3 and pathogenic bacteria in surprising ways that provide new insight into hyperglycemia-mediated immunocompromise contributing to bacterial infections in diabetic patients.

## Competing interests

The authors declare that they have no competing interests.

## Authors' contributions

PS conducted all functional and binding experiments, unless otherwise noted, and analyzed the results. CE conducted phagocytosis experiments and analyzed the results. RR contributed to the interpretation of results and editing of the manuscript. NK contributed to the interpretation of structural data and drafting or the manuscript. JN conducted the mass spectrometry experiments and analysis of these data. KC conceived of the study, coordinated design of the experiments, and drafted the manuscript with assistance as noted above. All authors read and approved the final manuscript.
